# Primary pulmonary epithelioid sarcoma: a case report

**DOI:** 10.1186/s13256-021-02940-0

**Published:** 2021-07-01

**Authors:** Eiki Mizutani, Riichiro Morita, Keiko Abe, Makoto Kodama, Shogo Kasai, Yasumi Okochi, Noriko Motoi

**Affiliations:** 1 Department of Thoracic Surgery, Tokyo Yamate Medical Center, 1-22-3, Hyakunin-cho, Tokyo, 169-0073 Japan; 2 Department of Pathology, Tokyo Yamate Medical Center, 1-22-3, Hyakunin-cho, Tokyo, 169-0073 Japan; 3 Department of Respiratory Medicine, Tokyo Yamate Medical Center, 1-22-3, Hyakunin-cho, Tokyo, 169-0073 Japan; 4 Department of Diagnostic Pathology, National Cancer Center Hospital, 5-1-1 Tsukiji, Chuo-ku, Tokyo, 104-0045 Japan

**Keywords:** Epithelioid sarcoma, Lung, Proximal-type, Neoplasms

## Abstract

**Background:**

Epithelioid sarcoma most frequently occurs in the dermal or subcutaneous area of the distal extremities. To date, there have been three cases of primary pulmonary epithelioid sarcoma reported. We report a case of epithelioid sarcoma that is considered a primary lung tumor.

**Case presentation:**

A 65-year-old asymptomatic Asian male patient underwent chest radiography during a routine health examination, and an abnormal mass was detected. His past medical history was unremarkable. He smoked 40 cigarettes every day and had slightly obstructive impairment on spirometry. He worked as an employee of a company and had no history of asbestos exposure. He underwent partial resection of the right lung by thoracoscopy. A histological examination of the tumor revealed a cellular nodule of epithelioid and spindle-shaped cells. Some of the tumor cells displayed rhabdoid features and reticular arrangement in a myxomatous stroma. Immunohistochemically, the tumor cells were positive for vimentin, smooth muscle actin (SMA), CD34, and epithelial membrane antigen (EMA); loss of the BAF47/INI1 protein in the tumor cells was also confirmed. A diagnosis of epithelioid sarcoma was established. Careful screening by whole-body positron emission tomography for another primary lesion after surgery did not detect any possible lesion. He had no cutaneous disease.

**Conclusion:**

To our knowledge, this is the fourth case of a proximal-type epithelioid sarcoma considered as a primary lung tumor.

## Background

Epithelioid sarcoma is a rare soft-tissue sarcoma. The tumor most frequently occurs in the dermal or subcutaneous area of the distal extremities of young adults, mostly males [1]. In 1997, Guillou *et al.* described proximal-type epithelioid sarcoma, which is found mainly in the pelvic and perineal regions and genital tracts of young to middle-aged adults and is characterized by a proliferation of epithelioid-like cells with rhabdoid features and the absence of a granuloma-like pattern [2].

We herein report a case of proximal-type epithelioid sarcoma considered a primary lung tumor. To our knowledge, this is the fourth case of primary pulmonary epithelioid sarcoma reported to date [3–5].

## Case presentation

**Fig. 1:**
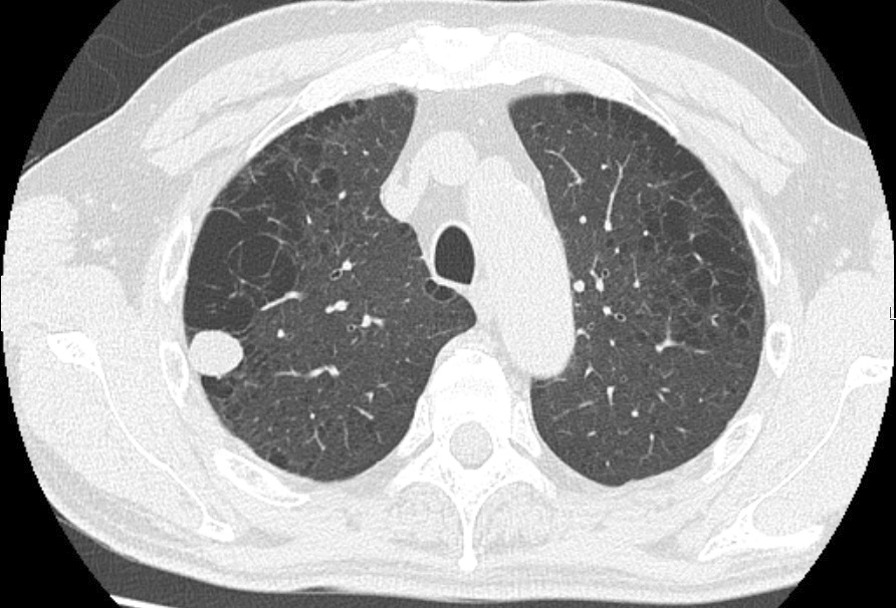
Chest computed tomographic scan showing a 2.0-cm soft-tissue nodule in the right upper lobe of the lung

A 65-year-old asymptomatic Asian male patient underwent chest radiography during a routine health examination, and an abnormal mass was detected. His past medical history was unremarkable. He smoked two packs of cigarettes per day for 45 years and had slightly obstructive impairment on spirometry. He worked as an employee of a company and had no history of asbestos exposure. Computed tomography showed two nodules in the right lung: a 1.5-cm soft-tissue nodule in the right upper lobe and a 0.5-cm soft-tissue nodule in the right lower lobe. After 3 months, the larger nodule had increased to 2.0 cm in diameter (Fig. 1), but the smaller nodule was unchanged.

**Fig. 2:**
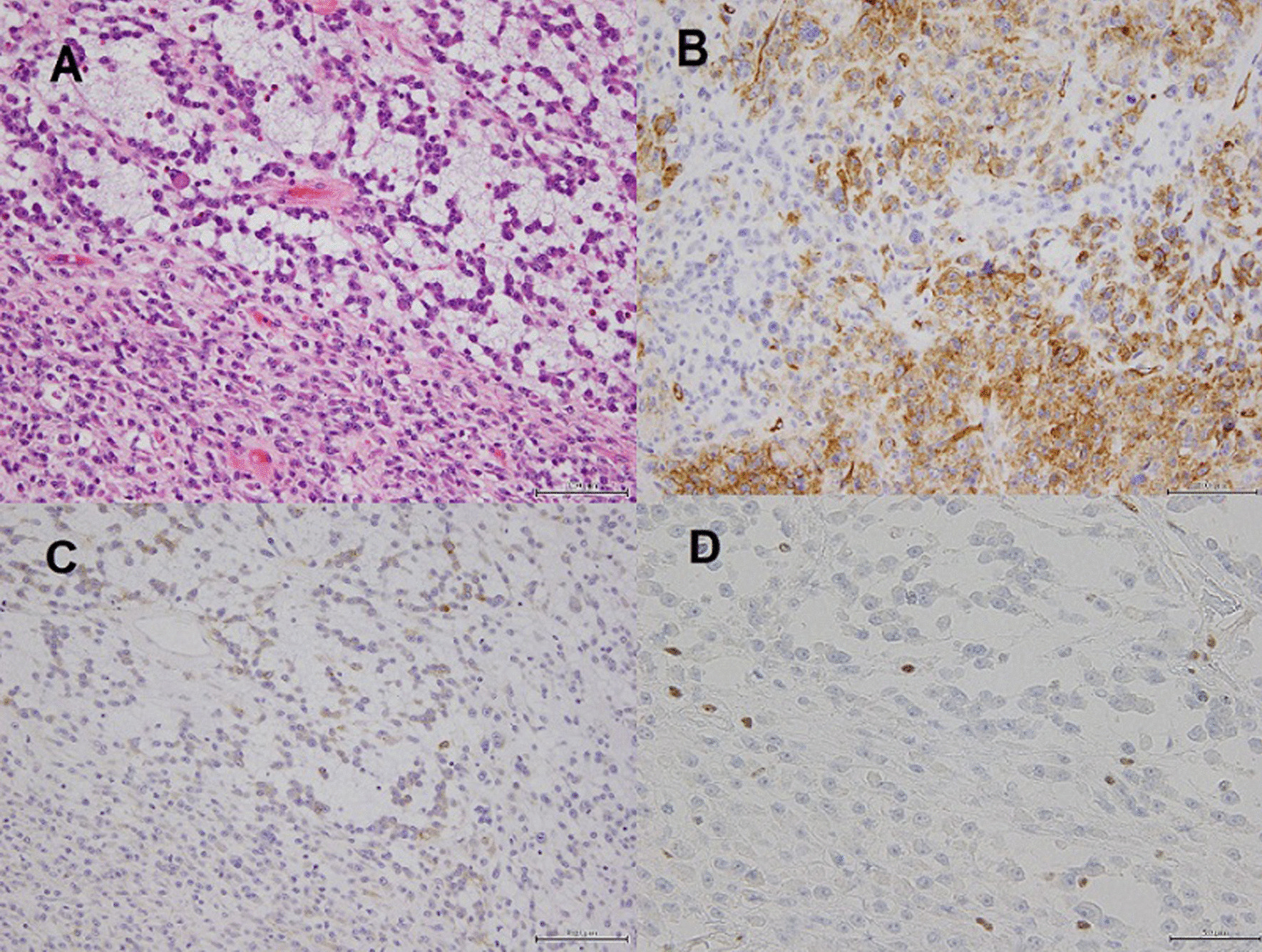
**A** Tumor cells showing spindle-shaped to epithelioid features with abundant eosinophilic cytoplasm, large vesicular nuclei, and prominent nucleoli (hematoxylin and eosin stain, ×200). **B** Anti**-**CD34 antibody positivity of the tumor cells (×200). **C** Epithelial membrane antigen positivity of the tumor cells (×200). **D** Loss of BAF47/INI1 protein in the tumor cells (×400)

The patient underwent wedge resection of the right upper and lower lobes by a standard three-port thoracoscopy. We used Endo GIA^TM^ Reinforced Reload with Tri-Staple Technology for the upper lobe with emphysematous changes; we used conventional cartridges for the lower lobe. There was no air leak during the operation, and the staple line was not reinforced. He had no air leakage, and we removed the chest drain on postoperative day 2. He was discharged uneventfully on postoperative day 7. A histological examination of the tumor in the upper lobe of the right lung revealed a cellular nodule of epithelioid and spindle-shaped cells. The tumor cells were arranged in solid sheets or fascicular arrangement and were loosely cohesive. Some of them exhibited rhabdoid features and reticular arrangement in a myxomatous stroma (Fig. 2A). Mitotic figures were sparsely observed. Immunohistochemically, the tumor cells were positive for vimentin, smooth muscle actin (SMA), CD34 (Fig. 2B), and endothelial membrane antigen (EMA) (Fig. 2C) and negative for desmin, erythroblast transformation-specific related gene (ERG), myoglobin, S-100 protein, HMB-45, melan-A, CD117, AE1/AE3, and CAM5.2. Loss of the BAF47/INI1 protein in the tumor cells was also confirmed (Fig. 2D). The lesion was pathologically diagnosed as epithelioid sarcoma. The surgical margins were negative. The nodule in the right lower lobe was histologically diagnosed as a hamartoma.

^18^F-fluorodeoxyglucose positron emission tomography performed on the 40th day after surgery showed no abnormal uptake in the whole body. He had no cutaneous disease. Follow-up chest roentgen at 4 months revealed no evidence of recurrence.

## Discussion and conclusion

We reported a case of epithelioid sarcoma that is considered a primary lung tumor.

Epithelioid sarcoma is a rare soft-tissue sarcoma. The tumor most frequently occurs in the dermal or subcutaneous area of the distal extremities of young adults, mainly in men [1]. It is a slow-growing neoplasm with a strong propensity for local recurrence and, ultimately, metastasis primarily to the lymph nodes, soft tissues, bones, lungs, and brain. Chase noted that the most common initial sites of metastasis are the lymph nodes (48%) and lungs (25%) [6].

In 1997, Guillou *et al.* described proximal-type epithelioid sarcoma as found mostly in the pelvic and perineal regions and genital tracts of young-to-middle-aged adults and tending to have an aggressive clinical course [2]. Proximal-type epithelioid sarcoma is characterized by a proliferation of epithelioid-like cells with rhabdoid features and the absence of a granuloma-like pattern [2]. To date, there have been three reported cases of primary pulmonary epithelioid sarcoma [3–5]. The first patient underwent radiation therapy and remained in remission for 78 months [3]. In the second case, pneumonectomy and adjuvant chemotherapy with ifosfamide plus doxorubicin were performed, and the patient remained in remission for 36 months after the diagnosis [4]. The third patient underwent chemotherapy and radiation therapy and died because of pulmonary metastasis and pneumonia 4 years after the initial treatment [5]. In the present case, the patient was treated with surgical resection without adjuvant chemotherapy, since the tumor was small and the role of systemic therapies in patients with epithelioid sarcoma is unclear [7]. This patient will need careful follow-up. To our knowledge, this is the fourth case of a proximal-type epithelioid sarcoma considered as a primary lung tumor. All four cases were male and past middle age, and the characteristics were the same as those of whole epithelioid sarcoma.

Histologically, epithelioid sarcoma tends to be characterized by predominantly epithelioid cells, marked cytological atypia, frequent occurrence of rhabdoid features, and absence of a granuloma-like pattern. In most cases, vimentin and cytokeratin are expressed [8, 9]. The BAF4/INI1 tumor suppressor gene is frequently inactivated in epithelioid sarcoma [10]. In our case, the lesion was diagnosed as an epithelioid sarcoma based on microscopic findings, an immunochemical examination, and loss of nuclear BAF47/INI1 expression in the tumor cells. Enzinger *et al.* reported that metastatic tumors differ from primary or recurrent tumors by a lesser degree of cellular differentiation and occasionally more extensive necrosis [1]. Distinguishing between a primary lesion and metastasis is difficult, but this lesion had only a small necrotic portion, and mitotic figures were sparsely observed. We considered the lesion to be a primary lesion based on the findings of ^18^F-fluorodeoxyglucose positron emission tomography and a medical examination by a dermatologist.

Due to the rarity of this tumor, there are limited data regarding the management of epithelioid sarcomas. Touati conducted a retrospective analysis of clinical data for epithelioid sarcoma patients and noted that the objective response and survival outcomes were similar between epithelioid sarcoma and nonselected sarcoma populations [7]. The current consensus suggests the performance of wide surgical resection and adjuvant chemotherapy, similar to the approach for managing soft-tissue sarcomas [1, 5].

## Data Availability

The datasets used and/or analyzed during the current study are available from the corresponding author on reasonable request.
